# A New Species of *Pachysentis* Meyer, 1931 (Acanthocephala: Oligacanthorhynchidae) in the Brown-Nosed Coati *Nasua nasua* (Carnivora: Procyonidae) from Brazil, with Notes on the Genus and a Key to Species

**DOI:** 10.2478/s11686-019-00080-6

**Published:** 2019-07-08

**Authors:** Ana Paula N. Gomes, Omar M. Amin, Natalie Olifiers, Rita de Cassia Bianchi, Joyce G. R. Souza, Helene S. Barbosa, Arnaldo Maldonado

**Affiliations:** 1 Laboratório de Biologia e Parasitologia de Mamíferos Silvestre Reservatório, Instituto Oswaldo Cruz, Fundação Oswaldo Cruz, Avenida Brasil, 4365 Manguinhos, Rio de Janeiro, RJ 21045-900 Brazil; 2 Pós Graduação em Biologia Parasitária, Instituto Oswaldo Cruz, Fundação Oswaldo Cruz, Rio de Janeiro, Brazil; 3 Institute of Parasitic Diseases, 11445 E. Via Linda 2‑419, Scottsdale, AZ 85259 USA; 4 Universidade Veiga de Almeida, Rua Ibituruna, 108 Maracanã, Rio de Janeiro, RJ 20271-020 Brazil; 5 Departamento de Biologia Aplicada à Agropecuária, Universidade Estadual Paulista “Júlio de Mesquita Filho”, Via de Acesso Prof. Paulo Donato Castellane s/n, Jaboticabal, SP 14884-900 Brazil; 6 Laboratório de Biologia Estrutural, Instituto Oswaldo Cruz, Fundao Oswaldo Cruz, Avenida Brasil, 4365 Manguinhos, Rio de Janeiro, RJ 21045-900 Brazil

**Keywords:** Acanthocephala, *Pachysentis lauroi* n. sp., Key to species, Carnivore, Mato Grosso do Sul, Brazil

## Abstract

**Introduction:**

*Pachysentis* comprises 10 species, which have been reported parasitizing mammals in Africa and the American continent. However, species of* Pachysentis* have not been described in brow-nosed coatis.* Pachysentis lauroi* n. sp. (Oligacanthorhynchidae: Acanthocephala) is described from the brown-nosed coati* Nasua nasua* (Linnaeus, 1766) Storr, 1780 (Procyonidae: Carnivora) in the Brazilian Pantanal wetlands of the Mato Grosso do Sul State, Brazil.

**Methods:**

Specimens were studied using light and scanning electron microscopy.

**Result:**

The new species is distinguished from other species of* Pachysentis* by the number of hooks in each longitudinal row (12 rows of 4 hooks, total of 48 hooks), presence of barbs on all hooks, and the organization of the cement glands. Notes on the genus* Pachysentis* [14] and a key to its species are provided. Critical comments on some species with a dubious diagnosis and questionable or missed key taxonomic characteristics are also reviewed. We also discuss the zoogeography of the members of the genus.

## Introduction

*Pachysentis* [14] comprises 10 species, which have been reported parasitizing mammals in Africa and the American continent [7–9, 13, 14, 16, 22, 23]. Acanthocephalans of wild Brazilian mammals have been studied mainly by Travassos [18–21] and Machado-Filho [12, 13], who described six species belonging to *Pachysentis,* five of these being reported in Brazil by Machado-Filho [13] and Vieira et al. [23]. These species are (1) *Pachysentis gethi* [13] [17] [syn. *Prosthenorchis gethi* [13] from *Eira barbara* (Linnaeus,1758) (Carnivora, Mustelidae) in Pará and Rio de Janeiro States and from *Galictis cuja* (Molina, 1782) and *G. vittata* (Schreber, 1776) in Rio de Janeiro [13, 16, 23]; (2) *Pachysentis procyonis* [13] [17] [syn. *Prosthenorchis procyonis* [13] from *Procyon cancrivorus* (Cuvier, 1798) (Carnivora, Procyonidae) in Rio de Janeiro State [13]; (3) *Pachysentis rugosus* [13] [17] [syn. *Prosthenorchis rugosus* [13] from *Sapajus cay* (Illiger, 1815) (Primates, Cebidae) in Rio de Janeiro State; (4) *Pachysentis septemserialis* [13] [17] [syn. *Prosthenorchis septemserialis* [13] from *Saguinus niger* (Hoffmannsegg, 1807) (Primates, Callitrichidae) in the Pará State [13]; Correa et al. [7]; (5) *Pachysentis lenti* [13] [17] [syn. *Prosthenorchis lenti* [13] from *Callithrix geoffroyi* (Humboldt, 1812) (Primates, Callitrichidae) in Espírito Santo State.

The brown-nosed coati *Nasua nasua* (Linnaeus, 1766) Storr, 1780 (Procyonidae) is a medium-sized carnivore abundant in many regions of South America [1, 5], especially in the Pantanal wetlands region [4, 5]. A few species of acanthocephalans have been reported infecting *N. nasua,* including *Oncicola luehei* [19, 17] in Pará, São Paulo, Minas Gerais, Mato Grosso, and Mato Grosso do Sul States [11, 13, 19, 23] and *Neoncicola potosi* [13, 17] in Foz de Iguaçú, Paraná State [15].

In this study, a new species, *Pachysentis lauroi* n. sp. is described using light microscopy and scanning electron microscopy (SEM) from the brown-nosed coati in the Brazilian Pantanal wetlands.

## Materials and Methods

Two adult brown-nosed coatis were found between 2007 and 2008 at the Nhumirin Ranch (18°59′S, 56°39′W), a research station of the Brazilian Agricultural Research Corporation (Embrapa/Pantanal) in the Nhecolândia subregion of the Pantanal, Mato Grosso do Sul State in the Brazilian Pantanal wetlands. The animals were collected during a research project investigating the ecology and health of wild carnivores. This research project included an inventory of helminth endoparasites. Acanthocephalan specimens were made available to parasitologists at the Oswaldo Cruz Foundation in Rio de Janeiro (FIOCRUZ/RJ). Animal procedures approved by the Brazilian Federal Environmental Agency (IBAMA, first license #183/2005, CGFAU/LIC; last license #11772-2) were followed.

The animals were necropsied and acanthocephalan specimens were collected from the small intestine of each individual host and stored in AFA (alcohol + formalin + acetic acid) for 24 h and stored in 70% alcohol. Worms used for microscopical studies were stained with acid (hydrochloric) carmine, dehydrated in a graded ethanol series, cleared in phenol 90% and mounted in Canada balsam (modified from [2], examined using an Axion Scope A1Light Microscope (Zeiss,Göttingen, Germany), and illustrated with the aid of a drawing tube attached to a Zeiss standard 20 light microscope (Zeiss, Göttingen, Germany).

Generic identification was based on the taxonomic key proposed by Schmidt [17] and specific taxonomic descriptions. The description of the new species of *Pachysentis* was based on 11 specimens (six males and five females). Measurements are in millimeters unless otherwise stated. The range was followed by the mean in parentheses. Proboscis hooks were counted in longitudinal alternating rows; hooks were measured in terms of its total length: from basal region of hook to the tip, length of the root, and were measured hook + root (tip of the hook to base of the root). The accepted species of *Pachysentis* deposited in the Coleção Helmintológica do Instituto Oswaldo Cruz—CHIOC (Helminthological Collection of the Oswaldo Cruz Institute), *P.gethi* [13, 17] (CHIOC 15680, 17836 a, 17837 b-d, 17838 a-b, 17846, 17852, 38100), *P.rugosus* [13, 17] (CHIOC 17827, 17828 b-c, 17848), *P.procyonis* [13, 17] (CHIOC 17847, 17833 a-b, 17854), *P.septemserialis* [13, 17] (CHIOC 10593, 17812 a-b), *P.lenti* [13, 17] (CHIOC 14830, 17819 a, 17820 a-c) and species deposited in the Museum für Naturkunde, Berlin, *P.procubens* [14] (No. 2440, 2443, 2474, 6032), *P.ehrenbergi* [14] (N°2426, 2432, 6033), *P.canicola* [14] (No. 2571) were used for comparison. Specimens of *Pachysentis lauroi* n. sp were deposited in the Helminthological Collection of the Institute Oswaldo Cruz (CHIOC), Rio de Janeiro, Brazil, under the number CHIOC no. 38565a (holotype) and 38565b (allotype).

For SEM, the specimens were fixed for 1 h at room temperature in 2.5% glutaraldehyde in 0.1 M Na-cacodylate buffer, washed in the same buffer and post-fixed for 3 h at room temperature in 1% osmium tetroxide in 0.1 M Na-cacodylate buffer. The material was then dehydrated in ascending ethanol series, critical point dried with CO_2_, mounted with silver cello tape on aluminum stubs, and sputter coated with a 20-nm-thick layer of gold. Samples were examined using a Jeol JSM-6390 LV microscope (JEOL, Akishima, Tokyo, Japan) at an accelerating voltage of 15 kV at the Electron Microscopy Platform of the Oswaldo Cruz Institute.

## Results

### Description

Order Oligacanthorhynchida Petrochenko, 1956

Family Oligacanthorhynchidae Southwell et Macfie, 1925

*Pachysentis lauroi* n. sp. (Figures 1–11)

**Fig. 1–5:**
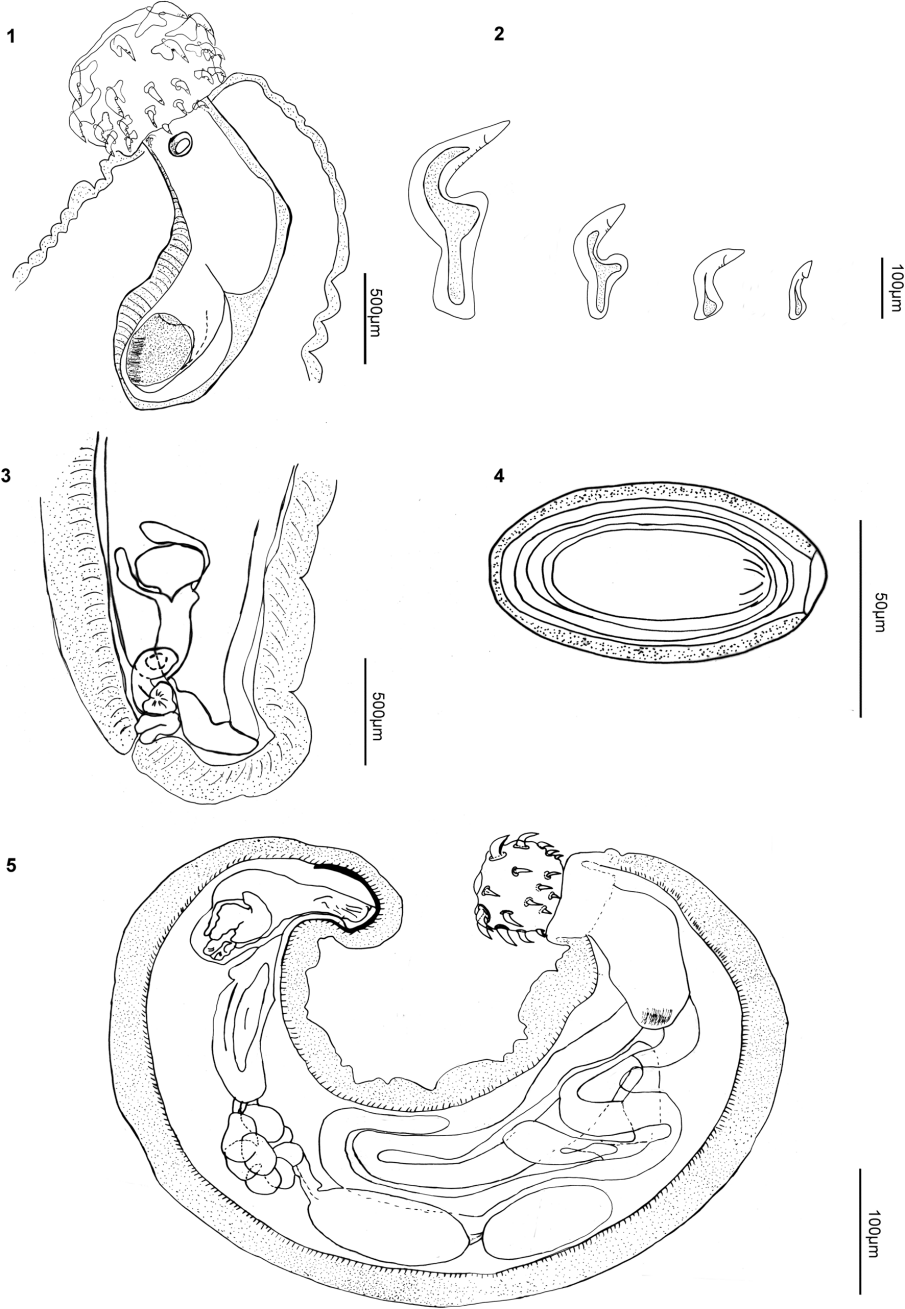
Line drawing of *Pachysentis lauroi* n. sp. collected in the intestine of *Nasua nasua* from the Brazilian Pantanal Wetlands, Mato Grosso do Sul State.** 1**—globular proboscis with hooks and proboscis receptacle with cephalic ganglion in proximal region;** 2**—row with four hooks, apical hooks with double root and proximal hooks with simple root;** 3**—posterior region of female showing the vagina, uterus and uterine bell;** 4**—ellipsoidal egg with three layers;** 5**—adult male showing two testes, “clustered” cements glands, ejaculatory ducts and retracted copulatory bursa

**Fig. 6–11:**
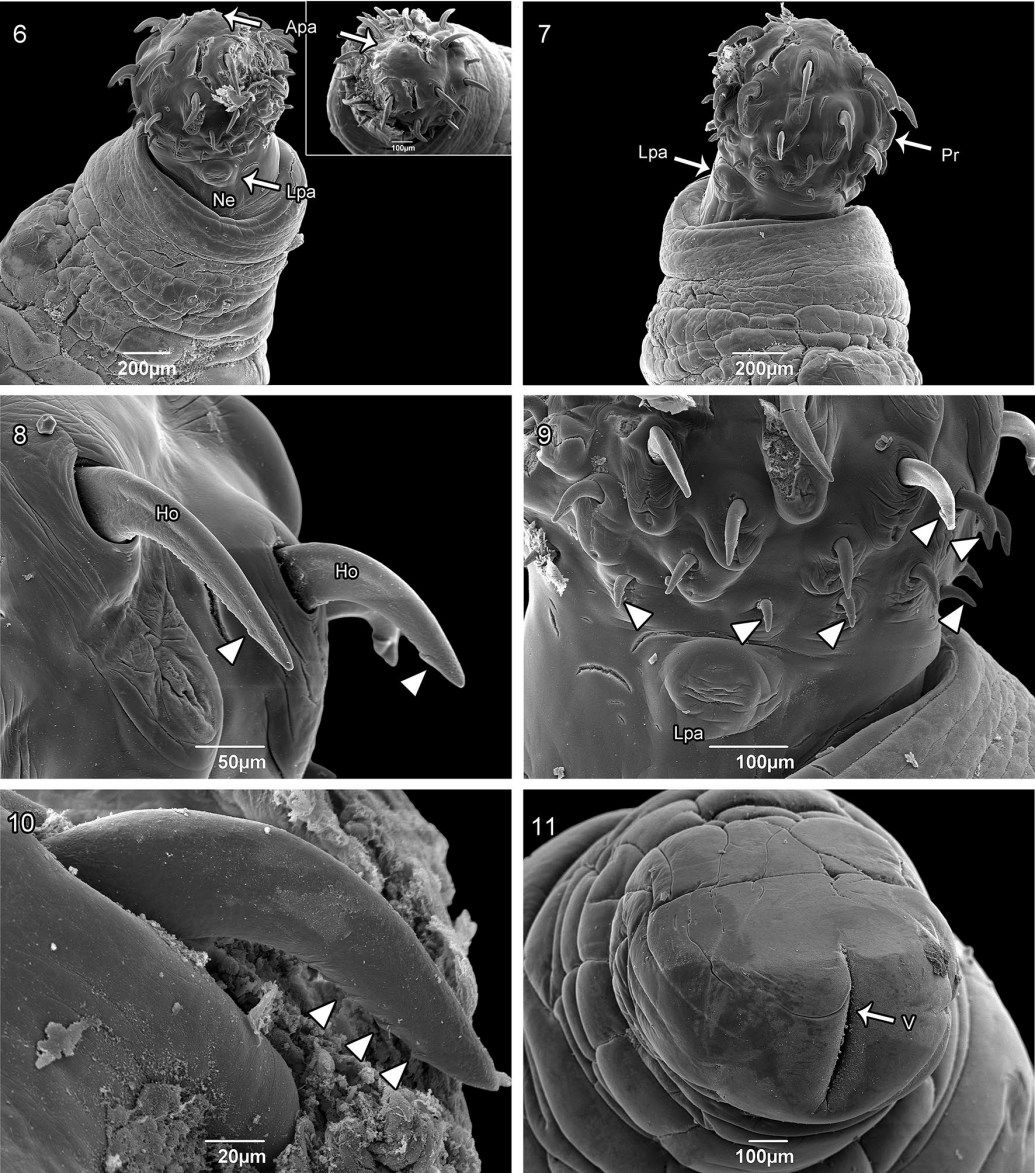
Scanning electron micrographs of specimens of* Pachysentis lenti* from* Nasua nasua* in the Brazilian Pantanal Wetlands, Mato Grosso do Sul State. **6** and **7**—globular proboscis with lateral papillae and apical papilla; **8** and **9**—apical and proximal hooks at base of the proboscis with barbs on the tips of the hooks (arrowhead); **10**—detail of the barbs on the tip of the apical hooks (arrowhead); **11**—posterior end of female body with subterminal vagina.* Lpa* lateral papillae,* Apa* apical papilla,* Ne* neck,* Pr* proboscis,* Ho* hook,* V* vagina

*General* With characters of *Pachysentis* is designated by Schmidt [17]. Trunk wider anteriorly. Proboscis subspherical with 12 longitudinal rows of four hooks each, totaling 48 hooks (Figs. 1, 2). Proboscis hooks similar in size and shape in both sexes. Apical hooks (types I and II) large with posterior curvature, complex manubria and double roots expanding laterally (Fig. 2). Proximal rows with short hooks (types III and IV) and simple discoid roots (Fig. 2). Measurements of length of apical and proximal hooks: length of hook × length of root and [length from proximal extremity to distal extremity in parentheses] in micrometers: (I) 150–229 (182) × 142–203 (170) [197–207 (249)]; (II) 97–145 (115) × 58–113 (81) [126–184 (153)]; (III) 45–118 (70) × 21–53 (39) [61–129 (91)]; (IV) 26–87 (53) × 18–39 (27) [39–103 (63)]. Hooks with terminal barbs visible by light microscopy in all types of hooks (Figs. 2, 8, 9, 10). Base of proboscis surrounded by lateral papillae with elevated border and central pore (Figs. 1, 6, 7); single apical papilla present with elevated border and salient tip at center (Fig. 6, insert). No marked neck. Proboscis receptacle similar in shape and size in both sexes, with two sub-regions measuring 0.87–1.33 (1.16) × 0.43–0.56 (0.47), with cephalic ganglion region (Fig. 1). Lemnisci long, flattened and curved (Fig. 5).

*Males* (based on six specimens): Trunk 6.00–16.61 (9.63) × 1.53–2.53 (1.91) wide anteriorly (Fig. 5). Proboscis 0.51–0.73 (0.64) × 0.68–0.85(0.73) wide. Lemnisci 4.75–6.83 (5.60), reaching middle of trunk (Fig. 5). Reproductive system in posterior 2/3 of trunk. Testes almost equatorial, contiguous, ellipsoid, in tandem (Fig. 5). Anterior testis 0.85–1.76 (1.15) × 0.32–0.62 (0.48); posterior testis 0.90–1.90 (1.27) × 0.48–0.60 (0.55) (Fig. 5). Eight compact uninucleate cement glands, 0.72–1.22 (0.86) × 0.44–0.68 (0.56). Ejaculatory duct 1.10–2.13 (1.42). Copulatory bursa terminal, retracted in all specimens (Fig. 5).

*Females* (based on five specimens): Trunk 10.79–12.95 (12.07) × 0.53–2.45 (1.62) anteriorly. Proboscis 0.53–0.87 (0.73) × 0.68–0.83 (0.78). Lemnisci 3.30 long in 1 specimen; others masked by eggs. Gonopore subterminal (Fig. 3). Vagina 0.16–0.21 (0.19) long (Figs. 3, 11); uterus 0.61–0.96 (0.80); uterine bell 0.23–0.38 (0.31) × 0.29–0.32 (0.30) (*n* = 2) (Fig. 3). Total reproductive system 1.11–1.34 (1.19) (*n* = 3). Eggs ellipsoidal, with sculptured outer membrane, 0.064–0.082 (0.073) × 0.054–0.036 (0.045) (*n* = 29) (Fig. 4).

### Taxonomic Summary

Type host: *Nasua nasua* (Linnaeus, 1766) Storr, 1780 (brown-nosed coati).

Type locality: Nhumirim Ranch (18°85′90S, 56°83′90 W), Mato Grosso do Sul State, Brazil.

Site of infection: Small intestine.

Etymology: The new species is named in honour of Dr. Lauro Travassos, who contributed greatly to our knowledge of the Brazilian Acanthocephala.

### Remarks

In this study, we identified the specimens obtained from *Nasua nasua* (Linnaeus, 1766) Storr, 1780 as belonging to the Oligacanthorhynchidae and *Pachysentis* due to the presence of a subspherical proboscis, anterior trunk wider than posterior, proboscis with 48 hooks in 12 longitudinal rows of four hooks each using [17]. In addition, Machado-Filho [13] considered the number of hooks on the proboscis and the size of the testes as the best characteristics for identifying and distinguishing species of the genus. *Pachysentis lauroi* n. sp. is compared with the other valid species of *Pachysentis* in Table 1 and further distinguished in the dichotomous key presented below.

**Table 1:** Morphometric comparison of species of the genus Pachysentis (measurements in mm)

Characteristcs/species	*P. angolensis*		*P.canicola (type species)*		*P.procumbens* (*juvenile*)		*P.ehrenbergi*		*P.rugosus*		*P.procyonis*	
Author	[9]		[14]		[14]		[14]		(Machado-Filho [13] [17]		(Machado-Filho [13] [17]	
Type host	*Canis adustus*		Dog [14]		*Vulpes vulpes*		*Vulpes vulpes; Naja haje*		*Sapajus cay*		*Procyon cancrivorus*	
Type locality	Angola, Africa		Brazil, South America		Argo, Egito, Africa		Egito, Africa		Rio de janeiro, Brazil		Rio de janeiro, Brazil	
Trunk	Male	Female	Male	Female	Male	Female	Male	Female	Male	Female	Male	Female
	17-23 × 3.5–4	34-48 × 4.8–5.5	15-28 × 4-8	20–26 × 5-11	6 × 1.25	6 × 1.25	25 × 4	26-29 × 6	25 × 3.5	32 × 3	20-30 × 2–3	25-35 × 2–3
Proboscis	0.55-0.63 × 0.70–0.82		0.57-0.80 × 0.57–0.85		0.55 × 0.55		0.8 × 0.9		0.564 × 0.694		0.697 × 0.716	
Total number of hooks	42		72		90		102		42		42	
Hooks per row	6 × 4 + 6 × 3		6 × 4 + 12 × 4*		6 × 7 + 6 × 8		6 × 9 + 6 × 8		6 × 4 + 6 × 3		6 × 4 + 6 × 3	
Barbs in hooks	No barbs		No barbs		No barbs		Barbs		No barbs		No barbs	
Proboscis receptacle	1.5		2		1.2		1.3		1.24 × 0.481		1.37 × 0.531	
Leminisci	5.8–6		7		–		7 × 0.8		4.64		3.64	
Anterior testis	2–3 × 0.9	–	2	–	–	–	3	–	1.57 × 0.697	–	3.01 × × 1.24	–
Posterior testis	2–4.3 × 1.0	–	2	–	–	–	3	–	1.69 × 0.664	–	3.15 × 1.07	–
Dimension of group of cement gland	3	–	3	–	–	–	7	–	2.02	–	3.56	–
Ejaculatory duct length	2.3	–	–	–	–	–	–	–	1.68	–	3.53	–
Uterine bell	–	–	–	3. 15–8.15	–	–	–	–	–	5.86	–	4.64
Eggs	–	0.09 × 0.043	–	0.07 × 0.045	–	–	–	0.07 × 0.05	–	–	–	0.071 × 0.042

Characteristcs/Species	*P.gethi*		*P.lenti*		*P.dollfusi*		*Pachysentis louroi n. sp.* (present study)			
Author	(Machado-Filho [13] [17]		(Machado-Filho [13] [17]		(Machado-Filho [13] [17]		Present study			
Type host	*Eira barbara*		*Callithrix geoffroyi*		*Eulemur fulvus* (syn. *Lemur fulvus*)		*Nasua nasua*			
Type locality	Pará and Rio de Janeiro, Brazil		Espirito Santo, Brazil		Madagascar, Africa		Mato Grosso do Sul, Brazil			
Trunk	Male	Female	Male	Female	Male	Female	Male	Female		
	10–15 × 1.0–2.5	15–25 × 1.5–3	15–20 × 1.0–2.5	20–25 × 2–2.5	50 × 4	50 × 4	9.63 × 1.91	12.07 × 1.62		
Proboscis	0.583 × 0.794		0.63 × 0.664		–		0.68 × 0.76			
Total number of hooks	42		48		48		48			
Hooks per longitudinal row	6 × 4 + 6 × 3		6 × 4 + 6 × 4		6 × 4 + 6 × 4		6 × 4 + 6 × 4			
Barbs in hooks	No barbs		No barbs		Barbs		Barbs			
Proboscis receptacle	1.07 × 0.498		1.32		–		1.16 × 0.47			
Leminisci	3.48		3.15		4.3–6.6		4.45			
Anterior testis	1.40 × 0.581	–	1.76 × 0.51	–	–	–	1.15 × 0.48	–		
Posterior testis	1.40 × 0.581	–	1.82 × 0.547	–	–	–	1.27 × 0.55	–		
Dimension of group of cement gland	1.54	–	2.98	–	–	–	0.86 × 0.56	–		
Ejaculatory duct length	4.64	–	–	–	–	–	1.42	–		
Uterine bell	–	5.56	–	1.41	–	–	–	1.19		
Eggs	–	0.084 × 0.054	–	–	–	0.08 × 0.05	–	0.073 × 0.045		

### The Status of *Pachysentis septemserialis* [13]

The specimens from CHIOC (17812 a-b and 10593) were carefully studied and it was observed that they exhibited some morphological characters not mentioned in the original description. The paratype (permanent slides CHIOC 17812 a-b) was not informative regarding the number of hooks, and a collar was observed at the base of the proboscis, suggesting affiliation with the genus *Prosthenorchis* [18]. The female paratype from CHIOC 10593 has 12 longitudinal rows of four hooks with total of 48 hooks, which contradicts the number of the hooks given in the original description (seven rows of seven hooks, total 49 hooks) with no collar at the base of the proboscis [13]. Additionally, there is a lack of some information on this species, such as the taxonomic and morphometric characters of adult males. Therefore, we suggest that the specimens designated as *P. septemserialis* [13, 17] may be synonymous with *P. lenti* [13, 17], as to the number of the hooks, other morphometric characteristics and the fact that both are parasites of primates of the family Callitrichidae. The taxonomy of this species needs to be revised.

### The Status of *Pachysentis ehrenbergi* [14]

Specimens of *Pachysentis ehrenbergi* deposited in the Museum für Naturkunde from *Vulpes vulpes* (No. 2426) and *Naja haje* (No. 2432, 6033) were also examined. Specimens from both hosts had barbs on the tip of all hooks, which was not mentioned by Meyer [14] in the original description. Other morphological characteristics, such as the number of hooks, short neck, the presence and size of nuclei in the leminisci and the reproductive organs agree with the original description.

*Pachysentis lauroi* n. sp. distinguished from the other species of *Pachysentis* by a combination of morphological characters, including the number of the hooks in each longitudinal row, the presence of barbs on the hooks and the arrangement of the cement glands (Table 1). The following key and Table 1 do not include *P. septemserialis,* because of its uncertain taxonomic status, but enable the new taxon to be distinguished from the other nine recognized species of the genus.

1. Proboscis with 12 longitudinal rows, alternating or not, of 3–4 hooks each…2
  -Proboscis with 12 alternating longitudinal rows of 7–9 hooks…9
2. Proboscis with a total of 42–48 hooks…3
  -Proboscis with a total of 72 hooks…*P. canicola* [14]
3. Proboscis with a total of 42 hooks…4
  -Proboscis with a total of 48 hooks…5
4. Cement glands in pairs…6
  -Cement glands clustered…7
5. Hooks with visible barbs (“arrow-shaped hook tip”)…8
  -Hooks without barbs… *P. lenti* [13] [17]
6. Parasite of carnivores in Africa…*P. angolensis* [9] [17]
  -Parasite of carnivores in the Americas … *P. gethi* [13] [17]
7. Very short lemnisci not reaching anterior testis. Parasites of carnivores …*P. procyonis* [13] Schmidt, [17]
  -Leminisci reaching anterior testis. Parasites of primates…*P. rugosus* [13] Schmidt, [17]
8. Cement glands in pairs …*P. dollfusi* [13] Schmidt, [17]
  -Cement glands in clusters…*P. lauroi* n. sp.
9. Proboscis 0.55 mm wide, with a total of 90 hooks without barbs … *P. procumbens* [14]
  -Proboscis 0.8–0.9 mm wide, with a total of 102 hooks with barbs … *P. ehenbergi* [14]

*Pachysentis lauroi* n. sp. is further distinguished from *P. angolesis, P. canicola, P. procumbens, P. ehrenbergi, P. gethi, P. procyonis* and *P. rugosus* by the number of hooks in each row, with 12 longitudinal rows of four hooks each, totaling 48 hooks (Table 1). Our specimens were similar to *P. lenti* and *P. dollfusi* in the number of hooks (48) on the proboscis. The new species can, however, be distinguished from *P. lenti* by having barbs on all hooks and from *P. dollfusi* by the organization of the cement glands (in cluster *vs* in uniform pairs), the size of trunk and the definitive host (Table 1). In addition, when Machado-Filho [13] described *P. dollfusi*, he indicated that this acanthocephalan infected a zoo animal in Brazil and that is native of Madagascar. Golvan [10], however, warned that the origin of this species might not have been Madagascar. Nevertheless, it is not known whether the species originates in Brazil or Madagascar.

## Discussion

Meyer [14] proposed *Pachysentis* with the type species *P.canicola* [14] from a domestic dog in Brazil. The same species was found infecting a gray fox *Urocyon cinereoargenteus* (Schreber, 1775) (Carnivora: Canidae) in the United States [6]. Two additional species, *P. ehrenbergi* [14] and *P. procumbens* Meyer, [14], were described from *Vulpes vulpes* (Linnaeus, 1758) in Egypt [14, 22], suggesting that species from this genus are parasites of carnivores (Order Carnivora).

Van Cleave [22] also studied acanthocephalan parasites from North American mammals and recorded *P. canicola* in the gray fox and the skunks *Mephitis mephitis mesomelas* (Lichtenstein, 1832), *Conepatus leuconotus* (Lichtenstein, 1832) and *Spilogale gracilis leucoparia* (Merriam, 1890), and recognized the three previous species of the genus. Yamaguti [24] revised the classification of the Acanthocephala and considered their geographic distributions, revised the diagnosis of the genus *Pachysentis* and followed the classification of Meyer [14] and Van Cleave [22] with three species in the genus.

Schmidt [17] revised the family Oligacanthorhynchidae and transferred six species of *Prosthenorchis* [18] to the genus *Pachysentis*, i.e., *P. dollfusi*, *P. gethi*, *P. lenti*, *P. procyonis*, *P. rugosus*, *P. septemserialis* and *P. angolensis* [syn. *Oncicola angolensis* [9]. *Pachysentis* [14] then included a total of 10 species based on morphological features, such as an anterior trunk wider than the posterior trunk; the absence of a festooned collar; a globular proboscis with 12 longitudinal rows of 3–12 hooks, totaling 42–102 hooks; larger anterior hooks with complex manubria and roots, as well as rootless posterior hooks; tips of the hooks with or without barbs; long and flattened lemnisci arranged a band; testes in tandem in the mid-trunk; eight compacted cement glands; and oval eggs with sculptured outer membranes (Yamaguti [17, 24].

According to this classification, the type hosts for species of *Pachysentis* are primates and carnivores with geographic distributions restricted to Africa and North, Central and South America [7–9, 13, 14, 16, 22, 23]. In the revisions by Golvan [10] and Amin [3], the authors updated the classification of the Acanthocephala and considered *Pachysentis* as including 10 valid species described by Meyer [14], Golvan [9] and Machado-Filho [13]. Therefore, the member species are *P. canicola, P. ehrenbergi, P. procumbens*, *P. angolensis*, *P. dollfusi*, *P. gethi*, *P. lenti*, *P. procyonis*, *P. rugosus* and *P. septemserialis*.

Our study provides details of* Pachysentis lauroi n. sp.* by scanning electron microscopy showing the presence of barbs on hooks in the proboscis, and the apical and lateral papillae-like structure on the proboscis, as morphological character to identify the new species. Furthermore, we are adding new information of morphology of two species,* P. septemserialis* and* Pachysentis ehrenbergi* and their status in the genus. Finally, we report a new definitive host in a new geographical area for the* Pachysentis* genus.
